# Global lessons for strengthening breastfeeding as a key pillar of food security

**DOI:** 10.3389/fpubh.2023.1256390

**Published:** 2023-08-22

**Authors:** Cecília Tomori

**Affiliations:** ^1^Johns Hopkins University School of Nursing, Johns Hopkins University, Baltimore, MD, United States; ^2^Johns Hopkins Bloomberg School of Public Health, Johns Hopkins University, Baltimore, MD, United States

**Keywords:** breastfeeding, food security, climate change, infant and young child feeding in emergencies (IYCF-E), COVID-19 pandemic, disasters, commercial milk formula marketing, health policy

## Abstract

Breastfeeding is identified as a central pillar of food security by the World Health Organization, however globally significant challenges remain in achieving breastfeeding targets for early initiation, exclusive breastfeeding for 6 months, and continued breastfeeding for 2 years and beyond. Inadequate support in health systems, poor maternity protections and workplace policies, and insufficient regulation of commercial milk formulas, among other barriers, continue to undermine this key pillar across nations. This paper highlights the central importance of breastfeeding for food security across diverse global settings by examining three case studies: Honduras, Pakistan and the USA. The cases highlight the complex layering and intersections of key challenges that threaten breastfeeding in the era of pandemics, the climate crisis, conflict and global inequality. Lessons drawn from these case studies, combined with additional insights, reinforce the importance of multisectorial collaboration to scale up investment in creating equitable, enabling environments for breastfeeding. These structural and systems approaches can successfully strengthen the breastfeeding ecosystem to ensure greater first food system resilience in the face of global crises, which compound maternal and infant vulnerabilities. Additionally, the cases add urgency for greater attention to prioritizing breastfeeding and incorporating IYCF-E protocols into disaster preparedness and management into the policy agenda, as well as ensuring that first food security is considered in energy policy. An integrated approach to policy change is necessary to recognize and strengthen breastfeeding as a pivotal part of ensuring food security across the globe.

## Introduction

Breastfeeding is a cornerstone of infant and young child food security and healthy development (1). Globally, over 800,000 deaths of children under 5 years of age could be prevented by following optimal breastfeeding practices of early initiation (within the first hour), exclusive breastfeeding for 6 months, and continuing to breastfeed with appropriate complementary foods for 2 years (2, 3). Breastfeeding is the optimal first food – it is readily available in response to the infants’ needs, it provides both necessary nutrition and hydration, protection from infectious and noncommunicable diseases, and provides a sophisticated communication system between mother and child (2). It comprises a complex “biopsychosocial system” that provides the basis for infant and young child development (2). While these facts are appreciated in global health, action to implement policies that enable breastfeeding have not kept apace (2). This negatively impacts the food security of millions of children worldwide with far reaching impacts for their own survival, health and development, as well as for future generations (1, 2, 4).

Globally less than half of newborns are put to breast within the first hour, and a similar proportion are breastfed exclusively under 6 months (2). The 2023 Lancet Breastfeeding Series explored both the global epidemiology of breastfeeding and what solutions are necessary to create an enabling environment so that all who wish to breastfeed can do so. The Series highlighted the importance of multisectorial collaboration and systems-based approaches to bring together the necessary components of this environment, from the health system to the workplace to marketing regulations (2, 5). The present paper builds on this framework to explore the layering of influences that can challenge breastfeeding as the foundation of first food security through three case studies from Honduras, Pakistan, and the USA.

The three cases were selected based on geographic, cultural and socioeconomic diversity, and because of available literature that demonstrates the intersecting influences on breastfeeding through the lens of food security. The cases highlight threats to breastfeeding as the basis of first food security in an era of rapid change, marked by the increasing impacts of the climate crisis, pandemics, global inequality and conflict. All three cases highlight the impacts of the COVID-19 pandemic, which disrupted maternity care services (6, 7) and increased food insecurity globally (8). Pandemics highlight the intersecting impacts of shocks to an interdependent global economic and food systems brought about by attempting to reduce viral transmission, as well as the impacts of climate change, since climate change fuels increasing risk of future pandemics (9). The cases also provide additional insights into how the increasing impacts of climate change intersect with the landscape of complex systems that shape the first food environment that are already marked by profound inequality. The experiences drawn from each case highlight how existing inequities across and within nations make first food systems vulnerable, and how these vulnerabilities accumulate in the context of multiple emergencies, disasters and conflict. As we plan for resilient first food systems in the 21 century and beyond, we must direct special attention to these intersections so that we can prevent and mitigate the compounding harms that arise when multiple vulnerabilities collide.

### Honduras

Honduras was the site of several key trials for programs that successfully increased breastfeeding, reduced the routine use of commercial milk formula (CMF) at hospitals, and reduced malnutrition during the 1980s through 2012 (10). Significant progress was made in the implementation of breastfeeding training and breastfeeding-friendly policies, leading to increased breastfeeding indicators in the country (10). Demographic Health Survey (DHS) data from 2011 shows that breastfeeding initiation was high at 96%, although timely initiation (within first hour) was much lower at 64, and 44% received prelacteal feeds (11). Prelacteal feeds are strongly associated with early supplementation, self-reported insufficient milk as well as premature breastfeeding cessation (12). Breastfeeding at 6 months was 85.8% and 75.7% at a year (11). However, exclusive breastfeeding (EBF) under 6 months was only 31.2% (11).

Recent research highlights the multiple, intersecting layers of influence that influence infant and young child food security in the context of COVID-19, climate change, and local and global political circumstances (13). Honduras has faced significant challenges prior to the pandemic, including high prevalence of poverty, food insecurity and malnutrition, with one fifth of children experiencing stunting in 2020 (8). These patterns vary substantially across regions, and disproportionately affect Indigenous populations. Existing challenges were amplified during the COVID-19 pandemic (13). High rates of unemployment (40%) and the majority of employment in the informal sector (80%) were coupled with reliance of remittances from migrant labor. Strains on labor meant reductions in income, and ability to purchase food. Supply chain disruptions also meant lower availability of food. Access to existing nutrition programs was also limited by measures to slow the transmission of the virus, such as closure of schools where food was distributed and restrictions on transportation, which made food accessible. This meant additional strain on families trying to meet their basic nutritional needs. Such challenges always place pressure on breastfeeding because women often have to prioritize seeking work to generate income and provide adequate food for their families and face limited ability to continue breastfeeding in many of these work settings, particularly in the informal sector, where most people are employed in Honduras (10, 14–16). Even when women can take advantage of maternity leave in the formal sector, leave costs are only partially born by the employer. This may contribute to the relationship of employment being associated with lower EBF (16). The COVID-19 pandemic has also disrupted maternity services globally, with contradictory guidance issued by Honduras that discouraged skin to skin contact but also endorsed WHO Early Newborn Care Practices, which include skin to skin contact (17). The impact of disruptions on breastfeeding has not yet been examined.

Another major source of pressure exacerbating stress and food insecurity on Honduran families is international and domestic conflict (13). Fertilizer prices rose during Russia’s invasion of Ukraine beginning 2022, further limiting the availability of food production, and highlighting the vulnerabilities of interconnected global supply chains. Moreover, internal conflict has undermined safety and security in Honduras, putting additional pressure on breastfeeding families seeking to secure wages and food for themselves their families, and on accessing breastfeeding support. Existing research highlights the high risk of maternal and child malnutrition in conflict settings as well as the stress of conflict, which – in the absence of adequate support – may lead to perceptions of insufficient milk and early breastfeeding cessation (18).

Climate change poses acute risks to first food security. Honduras is vulnerable to extreme weather events, which are accelerating in scale and frequency driven by the climate crisis (13). The country has been subject to floods, landslides, and drought. Critically, during the acute phase of the COVID pandemic in the fall of 2020, two Category 4 hurricanes hit Honduras, affecting nearly half the population and causing massive destruction. The hurricanes caused mass evacuations, and agricultural destruction which undermined the food supply, leading to insufficient supply and major price increases that made what was available inaccessible to many. The damage to roads and infrastructure made accessing food further problematic and also led to water contamination.

It was in this context that unsolicited CMF donations from wealthy settings started pouring into the country (13). Such donations violate the International Code of Marketing of Breast-Milk Substitutes and have been consistently linked to undermining breastfeeding and increasing diarrheal illness and malnutrition (19). Operational guidance on infant and young child feeding in emergencies (IYCF-E) (20) provides detailed discussion on how to ensure continued breastfeeding support. The guidance also provides resources on relactation, wet nursing, or donor human milk when infants are not breastfed, and appropriate purchase, distribution, and safe preparation of CMF when breastfeeding is not possible or available (20). Assistance can be appropriately channeled to support IYCF-E efforts with appropriate financial and technical assistance resources instead of harmful mass distribution of CMF. Efforts were made to stem these unsolicited donations by UNICEF and Pan-American Health Organization (PAHO) (13), but the above example demonstrates the particularly harmful influence of the CMF industry in the context of disaster circumstances. The example also draws attention to the importance of wealthy nations’ infant feeding norms and their misperception of necessities for safe infant feeding in emergencies in poorer settings, especially in this time of climate crisis.

### Pakistan

Pakistan provides another case study for the layered influences that shape the first food environment. Prior to the pandemic, Pakistan also had a high prevalence of food insecurity driven by poverty and even more acute malnutrition compared with Honduras, with significant regional variation and much higher prevalence of food insecurity in some regions (21). There are significant intergenerational elements of malnutrition, with mothers being chronically malnourished, which leads to poor nutrition during pregnancy, smaller babies at birth who are already at greater risk for poor growth, and mothers who may have difficulty caring for their babies (21). Without sufficient support for maternal nutrition, breastfeeding and nutritious complementary foods, the cycle of malnutrition continues. Based on data from the National Nutrition Survey of 2018, malnutrition was the leading cause of death among children under 5 years of age (~50%), with over 40% of children stunted and nearly a fifth experiencing wasting (17.7%) (22).

Breastfeeding has a key protective role in infant and child health in Pakistan. In a nationally representative survey, breastfeeding was associated with a 98% lower risk of neonatal mortality, 96% lower risk of infant mortality, and 94% lower risk of mortality among children under 5 years of age (23). From the latest data (2017), breastfeeding at six months and at 1 year was 86% and 71.2%, respectively, both of which represent declines over a 9-year period. At the same time, gains were made in exclusive breastfeeding, from 37.1% in 2006 to 47.5% in 2017 (24).

Previous work shows that work conditions are a major barrier to breastfeeding. For example, one study from Karachi (25) showed that after a 3-month paid maternity leave only 15% of employers offered breastfeeding breaks and few offered any breastfeeding support, such as a breastfeeding corner, nursery, refrigerator, or pump. A 2020 qualitative study carried out prior to the COVID-19 pandemic in a different setting of the rural district Matiari of Sindh (22), highlighted numerous additional barriers to exclusive breastfeeding. These included different work demands, since women were needed to carry out field labor, as well as lack of awareness of the importance of EBF, prelacteal feeds, perceived insufficient breastmilk and concerns about maternal malnutrition. The influence of CMF marketing are apparent even in this small study, where a mother who perceived her breastmilk to be insufficient was advised by a doctor to feed her baby formula, without providing her any support for addressing potential breastfeeding challenges. Such advice is common globally due to lack of adequate lactation training for healthcare providers (HCPs) combined with CMF marketing to HCPs who are considered authorities on infant feeding (2, 5). This kind of advice has particularly detrimental consequences in the context of poverty and malnutrition.

As in other settings, the COVID-19 pandemic also profoundly affected food security in Pakistan, with food insecurity doubling during the first year of the pandemic (26). These impacts disproportionately affected already poorer households, and those relying on wage-earning labor compared to those who relied on agriculture, which may have buffered their ability to secure food for their households. Pakistani guidance was supportive of skin to skin contact and breastfeeding during COVID-19 (17), however widespread disruptions to maternity services were reported (27). Limited literature indicates that those who had shorter hospital stays were more likely to breastfeed, pointing to inadequate breastfeeding support at the hospital (28), but it is difficult to interpret these findings without pre-pandemic comparative data.

Unfortunately, Pakistan faces significant impacts from climate change, which has further eroded food security. In 2022 the country faced historic floods, which left over a third of the country under water (29). This presented an immediate threat to life, affected 33 million people, and caused enormous destruction of crops and livestock as well as infrastructure, including health facilities. 7.6 million people were displaced, and many continue to face hunger and malnutrition (30). Basic health services have been profoundly disrupted, with devastating consequences for pregnant people and children in particular (29) – including impacts on breastfeeding support. Repairing roads, bridges, and other critical infrastructure remains ongoing. As of spring 2023, 10 million people remained without safe drinking water (31).

Ethnographic research (32) focusing on internally displaced people due to prior flooding events in 2015 has identified a number of protective factors as well as barriers to breastfeeding in these challenging circumstances. While breastfeeding was a culturally valued practice that was often supported by family and community networks, there was little formal support for it and there was pressure to introduce tea, other milks and foods early on. Even in the context of internal displacement, CMF was sold and encouraged by some to address infant crying, which was interpreted as a sign of hunger. This interpretation of infant behavior is a common reason globally for introducing CMF (33), but in the context of high rates of malnutrition and unsafe water, the use of CMF often has devastating outcomes. Participants (32) identified the lack of health services that support breastfeeding as a key problem – an interviewee started feeding her baby CMF because she felt that her milk was insufficient. This is a common perception during disasters, but one can be resolved through skilled support (19). Scholarship is still emerging on the impacts of the most recent floods, but the lack of adequate breastfeeding support identified is magnified because of the much larger scale of the 2022 floods.

In hopeful developments, Pakistan has passed a new maternity leave law in July 2023 that guarantees the right to take 6 months paid leave after the first child, and 4 and 3 months for the subsequent two children and provides one month of paternity leave as well (34). Additionally, the Provincial Assembly of Sindh – which includes the capital of Karachi – passed the Sindh Protection and Promotion of Breastfeeding and Young Children Nutrition Act 2023, which protects breastfeeding from commercial influence in health settings and promotes breastfeeding (20). These steps provide much-needed policies for protecting first food security in Pakistan.

### US

The US, a high-income country with high level of internal inequality, provides the third case for analysis. In recent decades, the US had achieved significant increases in breastfeeding indicators overall, with 83% initiating breastfeeding, 55.8% continuing to 6 months, and 35.9% at 1 year (35). However, exclusive breastfeeding rates as well as breastfeeding duration drop off sharply within a month, down to 24.9% by 6 months (35). This drop-off is driven primarily by the lack of paid leave, which sets the US apart from all other wealthy nations (15). Additionally, due to structural racism, significant racial and ethnic disparities persist in breastfeeding across the entire spectrum of indicators (36, 37). Existing inequities, the inadequate social safety net, and lack of paid leave also sets up a paradox whereby whose facing poverty and food insecurity may be less likely to breastfeed because they have to return to work quickly and face additional stressors (15, 38–40). Many work settings do not accommodate breastfeeding, especially among low wage and hourly workers or those who may face hostile work conditions, such as undocumented workers. Employment breastfeeding protections until recently only applied to select groups of workers – the PUMP Act, enacted in 2023, now grants many more workers protections and the right to breaks to express milk while at work although gaps still remain (e.g., in the airline industry) (41).

The US’s inequities are reflected in the representation of women and children served by governmental nutrition programs: over half of infants in the US are supported by the Special Supplemental Nutrition Program for Women, Infants, and Children (WIC) (42). Moreover, racial and ethnic minorities are overrepresented in WIC because of the aforementioned structural inequities (37, 38). WIC has invested significantly in increasing breastfeeding support and has achieved much higher breastfeeding initiation rates, rising from 48.3% in 2002 to 78.5% in 2017 (43). These rates, however, are lower than eligible non-participants enrolled in Medicaid (43). Previous work has shown that lower food secure WIC participants stop breastfeeding sooner than desired, with Black women stopping sooner because of a need or desire for others to feed their babies, while Hispanic mothers who faced food insecurity stop sooner than desired because they worry about the adequacy of their milk (38). These persistent inequities reinforce the need for policy interventions that enable breastfeeding and provide additional culturally appropriate support for racial minority populations (43, 44).

The COVID-19 pandemic caused major disruptions throughout the country, including access to employment and consequently housing, food, and the first food environment (37). Multiple governmental policy measures were put in place to mitigate these impacts, including expanded access to Medicaid, unemployment and eviction protections, and food aid supplementation (45). Worker protections from COVID itself, however, were relatively limited, which meant that significantly more poor, racial minorities suffered COVID deaths than white people, particularly early in the pandemic (46). These impacts affected the most marginalized families who were considered essential workers, including pregnant people and their newborns. There were also major disruptions in maternity services, including a period in spring of 2020 when mother-infant separation was recommended in contradiction to WHO guidance (47). This guidance was later reversed during the summer of 2020 (47). Separation is well-known to undermine breastfeeding (7), and coupled with lack of breastfeeding support, it had predictable negative effects on breastfeeding (48). Additionally, pregnant and lactating women faced delays in guidance on vaccination, therefore facing a lengthy period of additional risks from the SARS-COV-2 virus (47), leading to more cases of acute illness, which once again undermined the health of the mother and infant, and threatened their ability to stay together.

The US faced an additional disaster during the COVID pandemic, caused by corporate disregard for CMF safety regulations. In 2022, after reports of multiple cases of infant illness and deaths due to contaminated formula, Abbott shut down production at its largest facility, which produced approximately a quarter of the nation’s formula (37). For WIC participants, this meant a particularly acute crisis because WIC participants rely more on CMF and are restricted to a specific brand that their state has contracted with. Although these restrictions were eased, the supply remained low, and lactation support was not adequately scaled up. Many news stories depicted the plight of desperate parents seeking formula for their infants. Although some were able to seek lactation support or obtain milk from human milk banks or from informal human milk sharing networks, some turned to diluting formula or making it at home (49). Investigative journalists revealed that the shutdown was preceded by years of undermining regulations aiming to improve safety (37), and cases where formula contamination led to serious harm but were suppressed by aggressive legal strategies (50).

The impacts of the crisis were magnified for many across the nation who have faced chronic lack of access to safe water – situations that are magnified by the climate crisis. For instance, 48% of Native households living on reservations lack running water (51), and many cities, such as Flint, Michigan, have face lead contamination as instantiations of environmental racism and injustice (52). In 2022 Jackson, Mississippi, a city with predominately Black population, experienced an acute water crisis due to decades of racist neglect of water infrastructure coupled with flooding propelled by climate change (53, 54). The city continues to struggle. In these cases, both the formula supply and opportunities for safe preparation are threatened, but to date there is not sufficient investment in national or state efforts to scale-up breastfeeding support and IYCF-E that meet current and future needs. Although the formula crisis has abated, communities who face lower breastfeeding rates and lack adequate support remain vulnerable to the impacts of unsafe formula preparation and additional shocks due to future crises. This fundamentally undermines infant and young child food security.

## Discussion

Each of these cases highlights the importance of breastfeeding as a foundation of food security and health, as well as the complex intersections of multiple sectors and drivers of challenges that threaten breastfeeding in an era of pandemics and the climate crisis. As the recent 2023 Lancet Breastfeeding Series discussed, creating an enabling environment for breastfeeding requires collaboration and investment, bringing together policies and practices across health systems, workplaces both in the formal and informal sectors, and regulations that govern trade and marketing (2). Examining the importance of breastfeeding in food security highlights each of these domains as well as the broader context of social inequities which shape these systems (1).

The broader patterns of global social inequities across nations and inequities within nations, both linked to historical patterns of colonization and exploitation, structure the landscape in which breastfeeding exists (1, 4, 55, 56). It is these inequities that shape access to basic resources, such as housing, food, healthcare and work. For instance, underlying maternal malnutrition driven by poverty places newborns at increased risk of malnutrition and infection, and their mothers require additional support for their own health as well as in supporting breastfeeding. Food insecurity and work demands, coupled with a lack of supportive work environment often force women to leave their infants in others’ care so that they can provide for their families, which leads to early introduction of complementary foods and premature breastfeeding cessation. These impacts can be seen even in wealthy settings such as the US, due to internal inequities driven by structural racism. Existing inequities also shape access to resources and quality care that can mitigate these impacts and provide the support necessary to initiate and sustain breastfeeding. A key element of addressing underlying inequities is valuing care work (57), and the process of breastfeeding itself. The Mothers’ Milk Tool is an effort to provide a quantification of the economic value of breastfeeding and mothers’ milk in order to highlight the often-overlooked value of breastfeeding in the broader economy (58).

Shocks like the COVID-19 pandemic have exacerbated challenges to meet these basic needs, and disrupted maternity care services, which often undermined timely initiation of breastfeeding and ongoing breastfeeding support (7, 59, 60). The impacts of the pandemic were unequally distributed – disproportionately impacting already poor nations and those who are most socially marginalized within wealthier settings (61, 62). Importantly, pandemics are likely to accelerate with rising global temperatures and increasing intermingling of humans and other species due to habitat destruction (9), so these compounding effects must be taken into account as we plan for the future. We have an opportunity to learn from the lessons of the COVID-19 pandemic and implement pandemic preparedness protocols that follow WHO guidance and prioritize keeping mothers and infants together and the provision of breastfeeding support (63).

Climate change is the largest threat to food security overall and first food security in particular. Researchers have shown that the scale and diversity of impacts on food security alone has likely been underestimated (64), and the impacts on human life are complex, multiple, and accelerating (65). These impacts put existing progress towards creating enabling environments for breastfeeding at risk and threaten to undermine future efforts to scale up these efforts. Climate change exposes underlying weaknesses in systems and places infants who are already vulnerable at even greater risk, especially if they are mixed feeding or formula dependent. The formula supply itself can be quickly undermined, and safe preparation of formula often becomes impossible, leading to infection, dehydration and malnutrition. The impacts of climate change are profoundly unequal. While the extraction of fossil fuels is driven primarily by the consumption in high income countries in the Global North, the consequences are disproportionately borne by poor countries in the Global South. The Pakistan floods of 2022 illustrated these climate injustices on a very large scale (66), and Honduras and the US provide additional warning signals for policymakers. Ensuring that breastfeeding is prioritized during non-emergency times creates a greater climate resilience so that limited emergency resources can be directed appropriately for IYCF-E. Additionally, the implementation of IYCF-E policies must be enhanced. Driven partly by marketing efforts that have normalized CMF feeding as the baseline cultural practice, and the lack of appreciation for the importance of breastfeeding and safe infant feeding practices in emergencies, wealthier settings often lack knowledge about IYCF-E protocols (67). In the US, for instance, the Infant and Young Child Feeding in Emergencies Toolkit only became available last year (68), and much work remains to scale up IYCF-E support. In Honduras, the Global Nutrition Cluster and UNICEF Honduras have been collaborating to develop groundwork for an IYCF-E action plan which will form the basis of a national strategy (69), and Pakistan is continuing efforts to strengthen and implement its own IYCF-E strategy (70).

Unethical marketing and corporate misconduct are another throughline that tie the cases together. Unethical marketing undermines breastfeeding and makes it much more likely that infants become formula dependent (5). For instance, in the US years of undermining and subverting safety regulations to increase short-term profits led to the need to shut down production, which compromised supply, and compounded the impacts of unethical marketing (37). Unethical CMF marketing also contributes to climate change, driven by the greenhouse gas (GHG) emissions generated primarily from cow’s milk production (71). The estimated excess GHG generated by 6 months of CMF feeding for each child is 228–288 kg of carbon dioxide (71). Together, these impacts lead to negative health consequences across settings, that are more accentuated among poor nations and among those who are most marginalized within settings. While the rise of climate-driven disasters continues to disproportionately impact poorer countries, even those in wealthier settings become vulnerable to these impacts, once again with poorer and more marginalized groups bearing the majority of burdens. Donations of CMF in the context of emergencies further multiply these impacts. Indeed, corporate behavior of CMF companies parallels that of fossil fuel companies, which are at the root of the climate crisis, and have systematically engaged in merchants of doubt tactics to downplay and cast doubt on evidence to delay policy action (5, 72).

## Conclusion

In the face of extreme global challenges, including pandemics, the climate crisis, and global inequity and conflict, it is especially important to put breastfeeding at the center of policy action (73). Lessons drawn from the case studies in this paper reinforce the need to address underlying social inequities and multisectorial collaboration across health, work, and trade/marketing policies (2), with special attention to integrating breastfeeding and IYCF-E into disaster preparedness and emergency management (74). Additional efforts should be taken to integrate first food security considerations into energy policy, particularly in nations most responsible for fossil fuel consumption, and those that contribute to excess GHG via failure to regulate predatory CMF marketing (71). Concerted effort and political will are needed to ensure that breastfeeding is recognized and appropriately supported as a key pillar of food security.

## Author contributions

The author confirms being the sole contributor of this work and has approved it for publication.

## Conflict of interest

The author declares that the research was conducted in the absence of any commercial or financial relationships that could be construed as a potential conflict of interest.

## Publisher’s note

All claims expressed in this article are solely those of the authors and do not necessarily represent those of their affiliated organizations, or those of the publisher, the editors and the reviewers. Any product that may be evaluated in this article, or claim that may be made by its manufacturer, is not guaranteed or endorsed by the publisher.
